# Highly smooth and parameter independent obstacle avoidance method for autonomous vehicle with velocity-varying obstacle

**DOI:** 10.1371/journal.pone.0303160

**Published:** 2024-06-06

**Authors:** Nanxi Yi, Zhixian Liu, Xiaofang Yuan

**Affiliations:** 1 Huaihua University, Huaihua, Hunan, China; 2 Gachon University, Seongnam, South Korea; 3 Hunan University, Changsha, Hunan, China; TU Wien: Technische Universitat Wien, AUSTRIA

## Abstract

One of the primary challenges for autonomous vehicle (AV) is planning a collision-free path in dynamic environment. It is a tricky task for achieving high-performance obstacle avoidance with velocity-varying obstacle. To solve this problem, a highly smooth and parameter independent obstacle avoidance method for autonomous vehicle with velocity-varying obstacle (HSPI-OAM) is presented in this work. The proposed method uses the virtual collision point model to accurately design the desired acceleration, which makes the obtained path highly smooth. At the same time, the method gets rid of the dependence on parameter adjustment and has strong adaptability to different environments. The simulation is implemented on the Matlab-Carsim co-simulation platform, and the simulation results show that the path planned by HSPI-OAM has good performance for obstacle with acceleration.

## Section 1: Introduction

Autonomous driving technology has been greatly developed due to its own merits [1, 2], and obstacle avoidance is one of the key components of autonomous driving [3]. According to different environments, obstacle avoidance can be divided into static obstacle avoidance and dynamic obstacle avoidance.

In a dynamic environment, obstacles need to be dealt with. This is critical for autonomous vehicles, especially those traveling at high speeds in dynamically changing environments. For this, an efficient computer vision algorithm is proposed and used for speed and depth determination of obstacles [4]. For the environment perception in navigation, an obstacle detection method based on visual optical flow is proposed. This method uses the optical flow field constructed by image sequence to provide depth clues for obstacle detection, and realizes the online and real-time processing of the algorithm [5]. Obstacle trajectories in dynamic collision avoidance methods are often considered linear or known, but this limitation is not accurate in many real-world situations. An obstacle motion prediction method is proposed, which can be obtained by training LSTM neural network online [6].

The first step is to implement obstacle avoidance in a static environment. A parallel genetic algorithm based on graphics processing is proposed and the quasi-optimal solution can be found in time for a fast path planning [7]. In [8], an improved bat algorithm based on Cauchy disturbance and a logarithmic decreasing strategy is designed for a mobile robot, and the proposed method can significantly reduce the length of planned path. Zhu, X. H. improves the traditional D* Lite algorithm for multi-goal path planning and collision avoidance in unknown environments, and the issue of limited steering maneuverability during autonomous navigation is addressed by designing a safe distance and expanding the search range [9]. For real-time path planning of mobile robot, a navigation control method using an artificial potential field (APF) algorithm and a grey wolves optimisation (GWO) method is proposed, and the navigation free from any dead-end situation [10]. The above methods have achieved good performance in static environment, however, the moving of obstacle is completely ignored.

In order to implement obstacle avoidance in a dynamic environment, a strategy of trajectory planning and tracking is presented based on an artificial fish swarm algorithm (AFSA) [11]. Du Toit [12]] presents a strategy for planning robot motions in uncertain environments using the reasoning about future evolution and uncertainties of the states of the moving obstacles. In [13], An Elman neural network is proposed to compensate the effect of uncertainties between the dynamic robot model and the obstacles. Kim, C. J. [14] proposes an obstacle avoidance method in the position stabilization of the wheeled mobile robots using interval type-2 fuzzy neural network, and it is robust against uncertainties. In [15], a general formulation of a predictive and multirate reactive planning method for AV in dynamic environments with uncertainty is introduced. Malone N. [16] uses a stochastic reachable set-based potential field to improve the success rate of path planning. In [11–16], although dynamic obstacle avoidance is realized, the speed of obstacle is set to a specific value and ignores the acceleration of the obstacle. In fact, the speed of obstacle may change in real time, which poses a challenge to the performance of existing obstacle avoidance methods.

In terms of the smoothness of the road, a smooth and ergonomic optimal lane-change trajectory is planned for an obstacle that moves at low speed [17]. A two-stage control method is proposed for path planning in highway cruise mode [18]. In order to solve the local path planning in the structured road, a Regional-Sampling RRT (RS-RRT) algorithm based path planning method is proposed for obstacle avoidance, and the search efficiency of sampling is improved by the local biasing and Gaussian distribution sampling [19]. For the situation that vehicle tire friction may approach the limit in an unsmooth path, resulting in increased difficulty in path tracking and even instability of the vehicle, Liang et al. propose a variable speed method to design feasible speed and acceleration during path tracking to ensure that the vehicle will not reach the limit of tire friction [20].

In addition, using an improved artificial potential field, Zhang, Z. W. proposes a structured road-oriented motion planning framework for collision avoidance of AV [21]]. The calculation time is shortened by reducing the number of design variables of the optimal path in [22]. For low-moving AV, a path planning system is proposed through a supervisory control method based on a barrier function [23]. A novel path planning system is presented for dynamic obstacle avoidance [24], and the velocity is considered as constraint to obtain the optimal velocity. A model predictive path-planning controller with potential functions and vehicle dynamics terms is introduced [25]. In [26], a 3-D virtual dangerous potential field is constructed as a superposition of trigonometric functions of the road and the exponential function of obstacles, which can generate a desired trajectory for collision avoidance. Liu, J. C. presents a nonlinear model predictive control formulation for large-size autonomous ground vehicle with high centre of gravity at high speed [27]. Good dynamic obstacle avoidance performance can be achieved in [21–27], while they all depend on the parameters of the evaluation function, and the same parameters may not work well in different environments. Therefore, reducing the dependence on parameters is critical to the practicability of method.

Based on the analysis of the above research status, highly smooth and parameter independent obstacle avoidance method for autonomous vehicle with velocity-varying obstacle (HSPI-OAM) is proposed to adapt to the obstacle with complex motion, and the path planned by HSPI-OAM has high smoothness and low parameter dependence. The contributions of this work are summarized below:

- For obstacle with arbitrary acceleration, the HSPI-OAM can achieve high-performance obstacle avoidance for AV, which allows it to adapt to most dynamic environments.
- The proposed virtual collision point model accurately calculates the required acceleration, thus ensuring the high smoothness of the path, which is beneficial to the smooth running of the vehicle and the reduction of path tracking error.
- The performance of obstacle avoidance does not depend on parameter adjustment due to collision time can be determined to the appropriate value according to demand. This enhances the adaptability of the method to different environments.

Compared with current obstacle avoidance methods, such as [11–16], the proposed method implements obstacle avoidance for obstacles with more complex motions, at the same time, the planned path has high smoothness, and the planning process is less dependent on parameters. The remainder of this paper is organized as follows. In Section 2, the problem statement is described. Section 3 introduces the design of HSPI-OAM. The simulation and analysis are provided in Section 4. Finally, Section 5 gives the conclusion.

## Section 2: Problem statement

It may be difficult to achieve high-performance obstacle avoidance by ignoring changes in obstacle velocity. In order to address this challenge, two problems should be considered: (1) The smoothness of the path; (2) The dependency issues for parameter.

### The smoothness of path

The smoothness of path is critical to the smooth running of vehicle and the accuracy of path tracking. In general, the path planning problem is considered as [25, 28]:

$$\begin{array}{c} \text{min}\;J=\underset{u_{c},\epsilon }{\text{min}\;}\sum_{k=1}^{N_{p}}U^{t+k,t}+∥y^{t+k,t}-y_{des}^{t+k,t}{∥}_{Q}^{2}+\\ ∥u_{c}^{t+k-1,t}{∥}_{R}^{2}+∥u^{t+k-1,t}-u_{c}^{t+k-2,t}{∥}_{S}^{2}+∥\epsilon_{k}{∥}_{P}^{2} \end{array}$$ (1)

where *U*^*t*+*k*,*t*^ denotes the cost of obstacle, $∥y^{t+k,t}-y_{des}^{t+k,t}{∥}_{Q}^{2}$ is the cost for tracking the desired path, $u_{c}^{t+k-1,t}{∥}_{R}^{2}$ represents the cost of control inputs, $∥u^{t+k-1,t}-u_{c}^{t+k-2,t}{∥}_{S}^{2}$ is the cost of violation of control input and $∥\epsilon_{k}{∥}_{P}^{2}$ denotes the cost of slack variables. *Q*, *R*, *S* and *P* are the corresponding weight matrix. When the vehicle is far from the obstacle, the value of *U* is small, while the other penalty terms are relatively large, and the path does not change for obstacle avoidance. Only when the vehicle is close to the obstacle, the value of *U* will increase dramatically, and the path will change for obstacle avoidance. At this time, the time for obstacle avoidance is not much, and the change of path must be relatively drastic.

### The dependency issues for parameter

**Fig 1:**
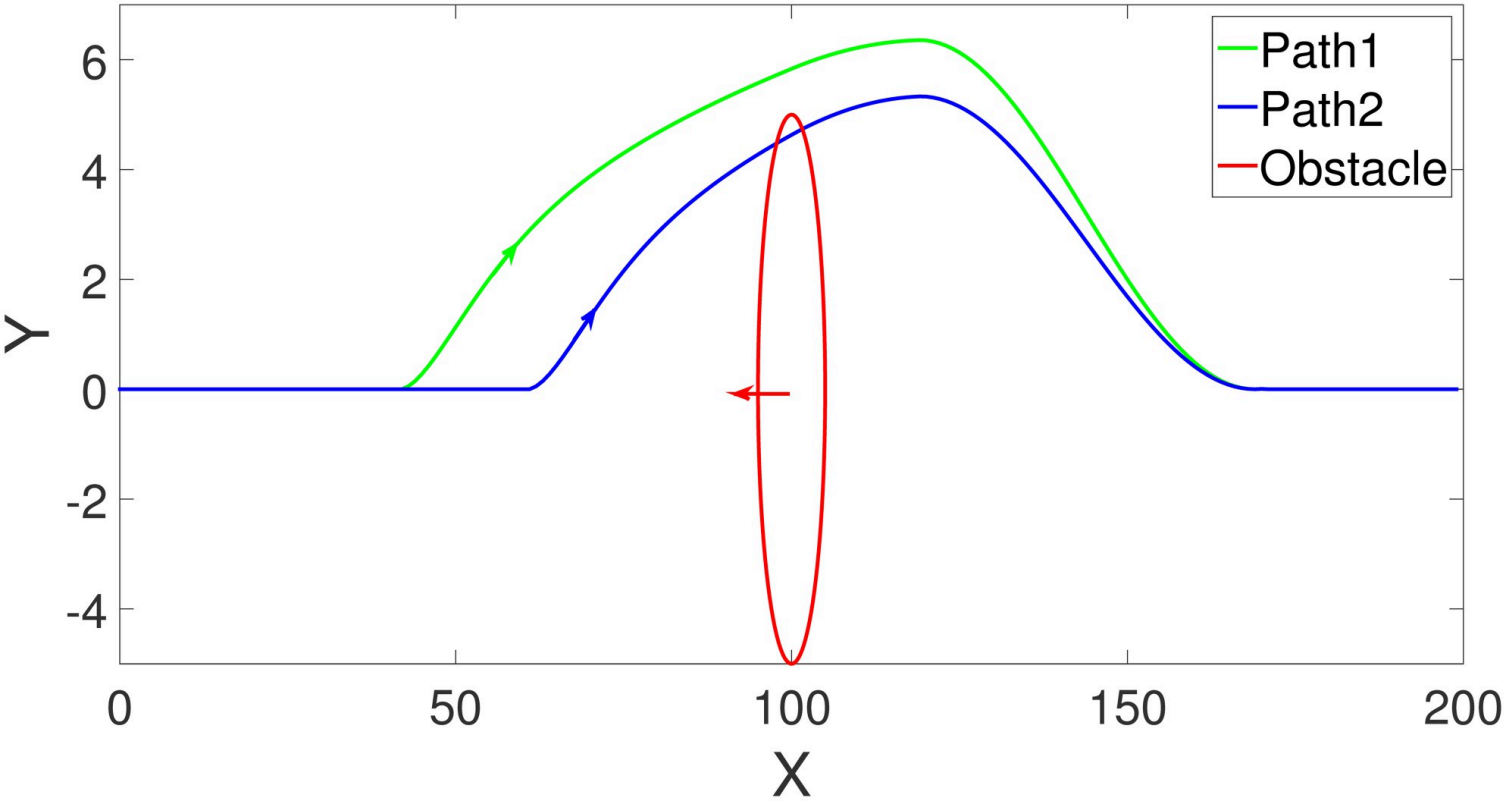
The obstacle avoidance with different parameters.

Generally speaking, the implementation of obstacle avoidance often depends on evaluation functions, and needs to evaluate a variety of objects, including obstacle avoidance, lane keeping, etc. These evaluation functions are usually scaled by weight coefficients. In dynamic environment, the same group of weight coefficients may not achieve good obstacle avoidance effect in the changing environment. In expressions (1), *U* is specifically expressed as:

$$\begin{array}{c} U=\frac{k_{1}}{s{\left(\frac{dX}{X_{s}},\frac{dY}{Y_{s}}\right)}^{k_{2}}} \end{array}$$ (2)

where *k*_1_ and *k*_2_ denote the intensity and shape parameters, respectively. $s(\frac{dX}{X_{s}},\frac{dY}{Y_{s}})$ is the signed distance which is consider the velocity of vehicle and obstacle, and more information about it can be found in [25]. In expressions (1) and (2), the cost of obstacle and the cost of other items have their respective coefficients. For specific environment, good obstacle avoidance effect can be achieved through adjusting these coefficients. However, when the velocity of obstacle is changed, the same parameters may not work well, or even cause obstacle avoidance to fail. The obstacle avoidance with different parameters is shown in Fig 1.

In Fig 1, the method proposed by [25] is used to avoid moving obstacles. The only difference between path 1 and path 2 is that *k*1 = 0.1 in path 1 and *k*1 = 0.01 in path 2. Obviously path 1 completes obstacle avoidance, while path 2 collides with the obstacle. The performance of obstacle avoidance depends on appropriate parameters. Usually in a dynamic environment, the same parameter is difficult to adapt to environmental changes.

## Section 3: The design of HSPI-OAM

**Fig 2:**
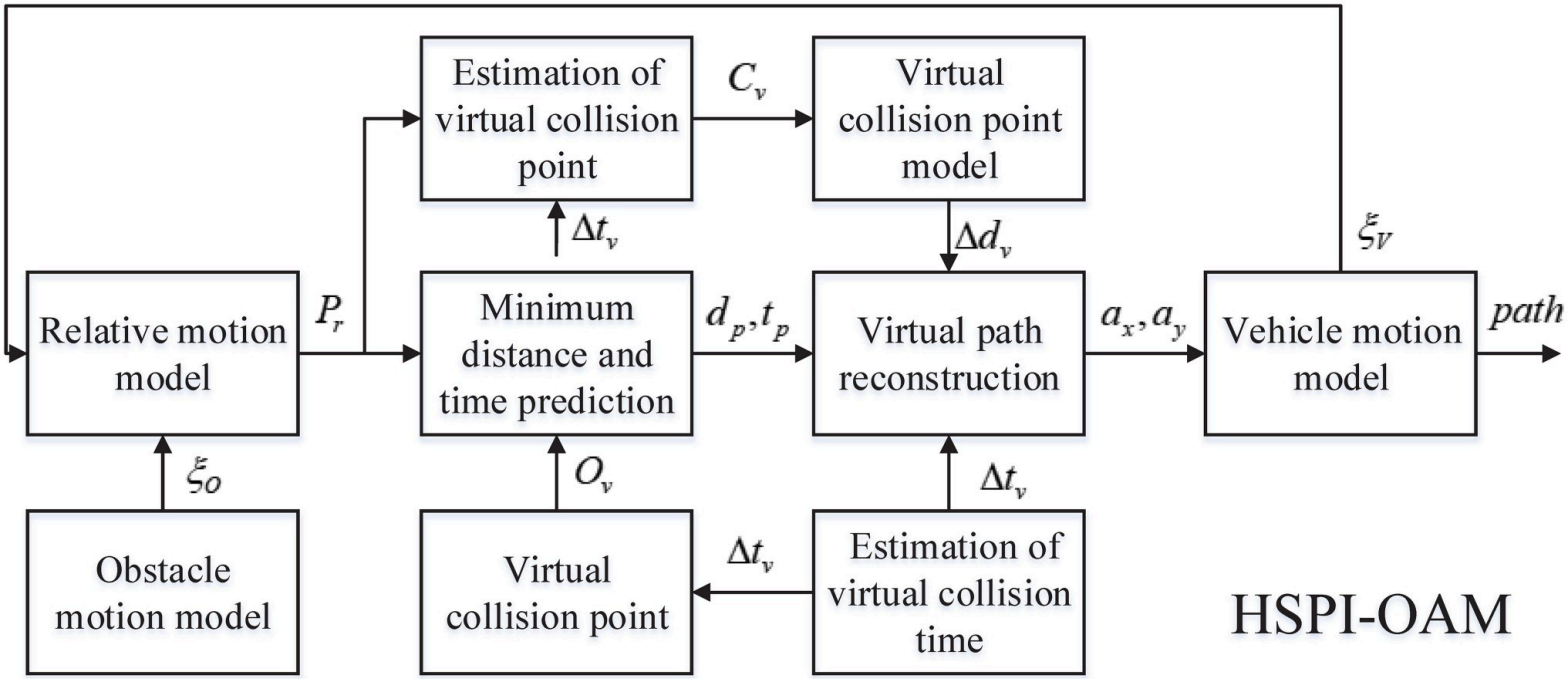
The structural schematic of the HSPI-OAM.

The existing methods ignore the acceleration of obstacle, and the avoidance maneuver is not timely and depend heavily on parameters. It may fail to achieve high-performance obstacle avoidance, or even lead to collision. To solve this problem, a highly smooth and parameter independent obstacle avoidance method for autonomous vehicle with velocity-varying obstacle (HSPI-OAM) is presented in this work. The structural schematic of HSPI-OAM is shown in Fig 2.

In Fig 2, *ξ*_*O*_ = [*a*_*ox*_ *a*_*oy*_ *v*_*ox*_ *v*_*oy*_ *x*_*o*_ *y*_*o*_]^*T*^ denotes the motion parameters of the obstacle, including its longitudinal acceleration, lateral acceleration, longitudinal velocity, lateral velocity, longitudinal position and lateral position. *ξ*_*V*_ = [*a*_*vx*_ *a*_*vy*_ *v*_*vx*_ *v*_*vy*_ *x*_*v*_ *y*_*v*_]^*T*^ is the motion parameters of the vehicle, including its longitudinal acceleration, lateral acceleration, longitudinal velocity, lateral velocity, longitudinal position and lateral position. *P*_*r*_ represents the predicted relative motion path. *d*_*p*_ is the minimum predicted distance between the vehicle and the obstacle, and *t*_*p*_ denotes the corresponding time. *a*_*x*_ and *a*_*y*_ are the average longitudinal and lateral accelerations required for obstacle avoidance, respectively. *O*_*v*_ represents the information of virtual obstacle. *C*_*v*_ denotes the information of virtual collision point. △*t*_*v*_ is the advance time for virtual collision. △*d*_*v*_ is the additional offset for avoiding collision. The relative motion model uses the motion parameters of vehicle and obstacle to obtain the predicted relative motion path. The minimum predicted distance between the vehicle and the obstacle and corresponding time can be predicted by combining the relative motion path and the information of virtual obstacle. In the virtual collision point model, the location of the virtual collision point is calculated using the time advance for virtual collision and the information of virtual obstacle. The virtual path is reconstructed by the input value to obtain the required average acceleration. The planned path and the motion parameters of vehicle at the next moment can be obtained from the vehicle motion model.

In order to visualize the problem, the moving obstacle is treated as stationary and the velocity of the moving obstacle is appended to the vehicle. For example, the velocity of the vehicle is *v*_*v*_, the velocity of the moving obstacle is *v*_*o*_, and after the obstacle is regarded as stationary and the velocity of the moving obstacle is attached to the vehicle, the velocity of the vehicle is *v*_*v*_-*v*_*o*_ and the velocity of the obstacle is regarded as 0. Under such conditions, the relative motion parameters of the vehicle are expressed as:

$$\begin{array}{c} \xi_{R}=\xi_{V}-\xi_{O}={\left[a_{rx}\;a_{ry}\;v_{rx}\;v_{ry}\;x_{r}\;y_{r}\right]}^{T} \end{array}$$ (3)

where [*a*_*rx*_ *a*_*ry*_ *v*_*rx*_ *v*_*ry*_ *x*_*r*_ *y*_*r*_]^*T*^ represent the motion parameters of the vehicle relative to the obstacle, including its longitudinal acceleration, lateral acceleration, longitudinal velocity, lateral velocity, longitudinal position and lateral position.

In order to improve the efficiency, the vehicle in this paper adopts the single point mass model considering acceleration [18]. And the relative motion model of the vehicle is expressed as:

$$\begin{array}{c} a_{rx}(k+1)=a_{rx}(k)+a_{x}(k) \end{array}$$ (4)

$$\begin{array}{c} a_{ry}(k+1)=a_{ry}(k)+a_{y}(k) \end{array}$$ (5)

$$\begin{array}{c} v_{rx}(k+1)=v_{rx}(k)+a_{rx}(k)\cdot t \end{array}$$ (6)

$$\begin{array}{c} v_{ry}(k+1)=v_{ry}(k)+a_{ry}(k)\cdot t \end{array}$$ (7)

$$\begin{array}{c} x_{r}(k+1)=x_{r}(k)+v_{rx}(k)\cdot t+0.5\cdot a_{rx}(k)\cdot t^{2} \end{array}$$ (8)

$$\begin{array}{c} y_{r}(k+1)=y_{r}(k)+v_{ry}(k)\cdot t+0.5\cdot a_{ry}(k)\cdot t^{2} \end{array}$$ (9)

where t represents sampling time interval. The predicted relative motion path can be obtained by the relative motion model. And the slope of the predicted relative motion path can be expressed as:

$$\begin{array}{c} k_{s1}=\frac{v_{ry}+a_{ry}\cdot t_{1}}{v_{rx}+a_{rx}\cdot t_{1}} \end{array}$$ (10)

Since the obstacle is considered stationary, *O*_*i*_ can be considered as the initial position of the obstacle, and it can be denoted as $O_{i}={\left[x_{o}^{0}\;y_{o}^{0}\right]}^{T}$, and the slope from *O*_*i*_ to *P*_*r*_ is:

$$\begin{array}{c} k_{s2}=\frac{y_{o}^{0}-(y_{r}+v_{ry}\cdot t_{1}+0.5\cdot a_{ry}\cdot t_{1}^{2})}{x_{o}^{0}-(x_{r}+v_{rx}\cdot t_{1}+0.5\cdot a_{rx}\cdot t_{1}^{2})} \end{array}$$ (11)

When the distance from *O*_*i*_ to *P*_*r*_ is the smallest, there is:

$$\begin{array}{c} \;\;\;\;\;\;\;\;\;\;\;\;\;\;\;\;\;\;\;\;\;\;\;\;\;\;\;\;\;\;k_{s1}\cdot k_{s2}=-1\;\;\;\;\;\;\;\;s.t.(t_{1}>=0) \end{array}$$ (12)

**Fig 3:**
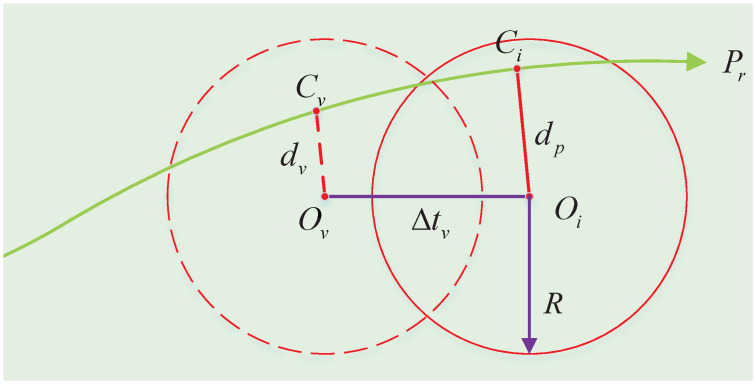
The virtual collision point model.

When expression (12) is true, *d*_*p*_ and *t*_*p*_ can be obtained. Then the virtual collision point model can be constructed as shown in Fig 3.

In general, if *d*_*p*_ is less than the collision radius *R* of the obstacle, the path needs to be offset outward by *R* − *d*_*p*_ along *d*_*p*_ in order to avoid collision. This will increase the lateral offset after obstacle avoidance because of the lateral acceleration. In order to reduce the lateral offset, *O*_*v*_ is simulated to collide with the vehicle in advance, and the advance collision time is △*t*_*v*_. In the virtual collision point model, *d*_*v*_ is expected to be obtained. The coordinate of *C*_*i*_ is expressed as:

$$\begin{array}{c} x_{ci}=x_{r}+v_{rx}\cdot t_{g}+0.5\cdot a_{rx}\cdot t_{g}^{2} \end{array}$$ (13)

$$\begin{array}{c} y_{ci}=y_{r}+v_{ry}\cdot t_{g}+0.5\cdot a_{ry}\cdot t_{g}^{2} \end{array}$$ (14)

The coordinate of *C*_*v*_ is expressed as:

$$\begin{array}{c} x_{cv}=x_{r}+v_{rx}\cdot (t_{g}-△t_{v})+0.5\cdot a_{rx}\cdot {\left(t_{g}-△t_{v}\right)}^{2} \end{array}$$ (15)

$$\begin{array}{c} y_{cv}=y_{r}+v_{ry}\cdot (t_{g}-△t_{v})+0.5\cdot a_{ry}\cdot {\left(t_{g}-△t_{v}\right)}^{2} \end{array}$$ (16)

The coordinate of *O*_*v*_ is expressed as:

$$\begin{array}{c} x_{ov}=x_{cv}-\left(y_{cv}-y_{o}^{0}\right)*\left(x_{ci}-x_{o}^{0}\right)/\left(y_{ci}-y_{o}^{0}\right) \end{array}$$ (17)

$$\begin{array}{c} y_{ov}=y_{o}^{0} \end{array}$$ (18)

Then *d*_*v*_ can be calculated as:

$$\begin{array}{c} d_{v}=\sqrt{{\left(y_{ov}-y_{cv}\right)}^{2}+{\left(x_{ov}-x_{cv}\right)}^{2}} \end{array}$$ (19)

In the virtual collision point model, additional offset △*d*_*v*_ is needed to avoid collision.

$$\begin{array}{c} △d_{v}=R-d_{v} \end{array}$$ (20)

And the additional offset needs to be done in (*t*_*p*_ − △*t*_*v*_) time. Therefore, the acceleration required is:

$$\begin{array}{c} a_{v}=\frac{2\cdot △d_{v}}{{\left(t_{p}-△t_{v}\right)}^{2}} \end{array}$$ (21)

The corresponding longitudinal and lateral acceleration components are:

$$\begin{array}{c} a_{vx}=a_{v}\cdot \text{cos}(\theta ) \end{array}$$ (22)

$$\begin{array}{c} a_{vy}=a_{v}\cdot \text{sin}(\theta ) \end{array}$$ (23)

$$\begin{array}{c} \theta =atan2(y_{ov}-y_{cv},x_{ov}-x_{cv}) \end{array}$$ (24)

where *atan*2 is an extension of the arctangent function, and it returns value in the range (−*pi*, *pi*]. The expression (15)—(22) is subject to *t* ∈ [0, *t*_*v*_ − △*t*_*v*_]. When *t* ∈ (*t*_*v*_ − △*t*_*v*_, *t*_*v*_), the expected accelerations are:

$$\begin{array}{c} a_{vx}=\frac{v_{refx}-v_{rx}}{△t_{v}} \end{array}$$ (25)

$$\begin{array}{c} a_{vy}=\frac{v_{refy}-v_{ry}}{△t_{v}} \end{array}$$ (26)

where *v*_*refx*_ and *v*_*refy*_ denote the longitudinal and lateral reference velocity of vehicle.*a*_*vx*_ and *a*_*vy*_ are the accelerations required for relative motion, and the actual acceleration components required by the vehicle are:

$$\begin{array}{c} a_{x}=a_{vx}+a_{rx} \end{array}$$ (27)

$$\begin{array}{c} a_{y}=a_{vy}+a_{ry} \end{array}$$ (28)

Finally, the high-performance path for obstacle avoidance and the vehicle motion parameters at the next moment can be obtained using the vehicle motion model.

It should be pointed out that the proposed method is planned at the acceleration level and the acceleration is equalized in the virtual collision point model, so the smoothness of the planned path is greatly improved.

## Section 4: Simulation and analysis

The performance of HSPI-OAM is tested by four comparative simulation tests, and improved artificial potential field method (IAPF) is used as the contrast method. The IAPF method uses the combination of artificial potential field and model prediction, both of which are classical path planning methods. Especially, the combination of the two methods makes the comparison method have strong dynamic planning ability by using prediction ability. Meanwhile, the comparison method can consider the actual physical constraints of vehicles in the model prediction, so that the path planned by the comparison method is smoother. Therefore, it is more comparable with the method proposed in this paper. In IAPF method, the velocity of obstacle is taken into account and the cost function is designed as expressions (1) and (2). More information about the IAPF method can be found in [28]. The result of test is summarized in Table 1. *β*_*max*_ is maximum side slip angle of vehicle. *γ*_*max*_ denotes the maximum yaw rate of a vehicle. *e*_*max*_ represents the maximum tracking error of vehicle. Generally speaking, after completing obstacle avoidance, the vehicle needs to return to the global path. At this time, the values of *β*_*max*_, *γ*_*max*_ and *e*_*max*_ can be reduced through parameter settings. For example, more time and distance can be used to make the regression path smoother, so as to reduce the above variables. Therefore, the *β*_*max*_, *γ*_*max*_ and *e*_*max*_ in Table 1 refer to the values before obstacle avoidance. The partial parameter of test is set in Table 2. *ξ*_*O*_ and *ξ*_*V*_ are the motion parameter of obstacle and vehicle as described in Fig 2. △*t*_*v*_ is the advance time for virtual collision as described in Fig 2. *Q* and *k*_1_ are the relative weight coefficient as described in expressions (1) and (2). In the proposed method, only △*t*_*v*_ needs to be determined. This parameter is first given an approximate value through experience, and then a better value is determined through multiple simulation tests.

**Table 1:** The result of tests.

Test	Method	Collision avoidance result	Minimum distance	∣*β*_*max*_∣	∣*γ*_*max*_∣	∣*e*_*max*_∣
Test1	HSPI-OAM	No collision	6.5m	0.13^∘^	0.94^∘^/s	0.01m
	IAPF	No collision	5.1m	1.08^∘^	7.79^∘^/s	0.21m
Test2	HSPI-OAM	No collision	6.2m	0.08^∘^	1.08^∘^/s	0.03m
	IAPF1	No collision	6.9m	0.67^∘^	7.81^∘^/s	0.36m
	IAPF2	No collision	7.0m	0.51^∘^	5.88^∘^/s	0.51m
Test3	HSPI-OAM	No collision	5.6m	0.95^∘^	2.01^∘^/s	0.05m
	IAPF1	Collision	2.7m	2.11^∘^	3.89^∘^/s	0.08m
	IAPF2	No collision	6.5m	2.07^∘^	3.87^∘^/s	0.08m
Test4	HSPI-OAM	No collision	5.8m	0.19^∘^	2.35^∘^/s	0.06m
	IAPF1	Collision	2.9m	0.32^∘^	3.76^∘^/s	0.15m
	IAPF2	No collision	6.3m	0.29^∘^	3.52^∘^/s	0.13m

**Table 2:** The parameter of tests.

Test	Motion parameters	Method	Performance parameters
Test1	*ξ*_*O*_(1) = [−1, 0, 0, 0, 150, 0]^*T*^	HSPI-OAM	△*t*_*v*_=2
	*ξ*_*V*_(1) = [0, 0, 10, 0, 0, 0]^*T*^	IAPF	*k*_1_=0.1,*Q*=0.001
Test2	*ξ*_*O*_(1) = [−2, 0, −10, 0, 300, 0]^*T*^	HSPI-OAM	△*t*_*v*_=2
		IAPF1	*k*_1_=0.1,*Q*=0.001
	*ξ*_*V*_(1) = [0, 0, 30, 0, 0, 0]^*T*^	IAPF2	*k*_1_=0.0015,*Q*=0.001
Test3	*ξ*_*O*_(1) = [0.5, 1, −17.5, −15, 208, 112.5]^*T*^	HSPI-OAM	△*t*_*v*_=2
		IAPF1	*k*_1_=0.1,*Q*=0.001
	*ξ*_*V*_(1) = [0.5, 1, 10, 0, 0, 0]^*T*^	IAPF2	*k*_1_=1.5,*Q*=0.001
Test4	*ξ*_*O*_(1) = [−0.125, 0.25, 14.15, 1.7, 100, 5.78]^*T*^	HSPI-OAM	△*t*_*v*_=2
		IAPF1	*k*_1_=1.5,*Q*=0.001
	*ξ*_*V*_(1) = [−0.5, 1, 30, 0, 0, 0]^*T*^	IAPF2	*k*_1_=50,*Q*=0.001

### Test 1

**Fig 4:**
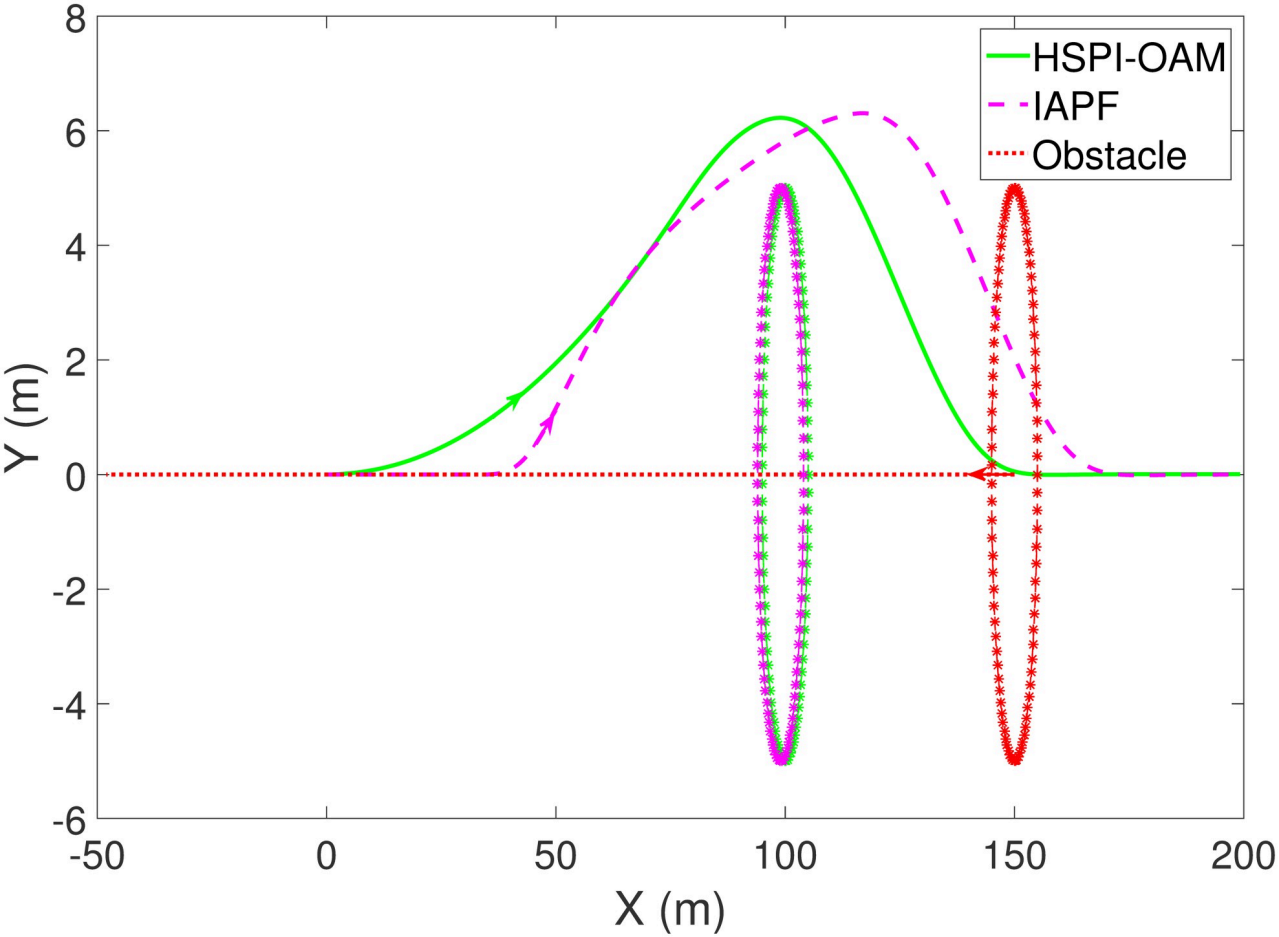
The trajectory of AV and obstacle of Test 1.

**Fig 5:**
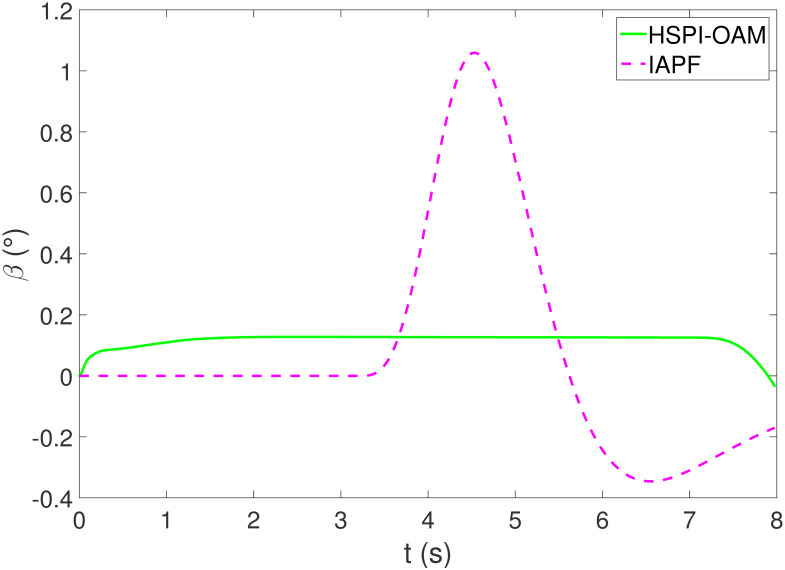
The side slip angle of Test 1.

**Fig 6:**
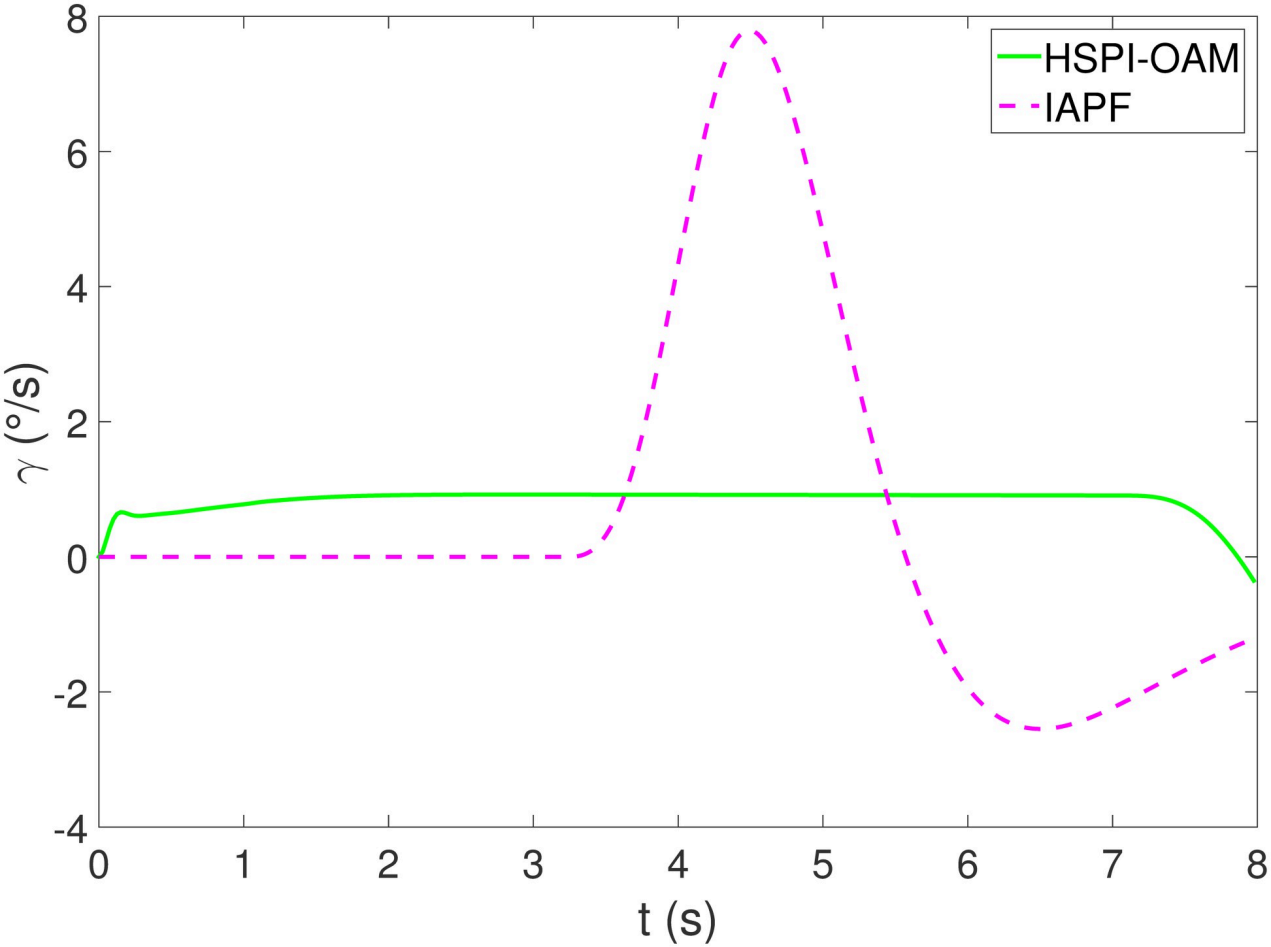
The yaw rate of Test 1.

**Fig 7:**
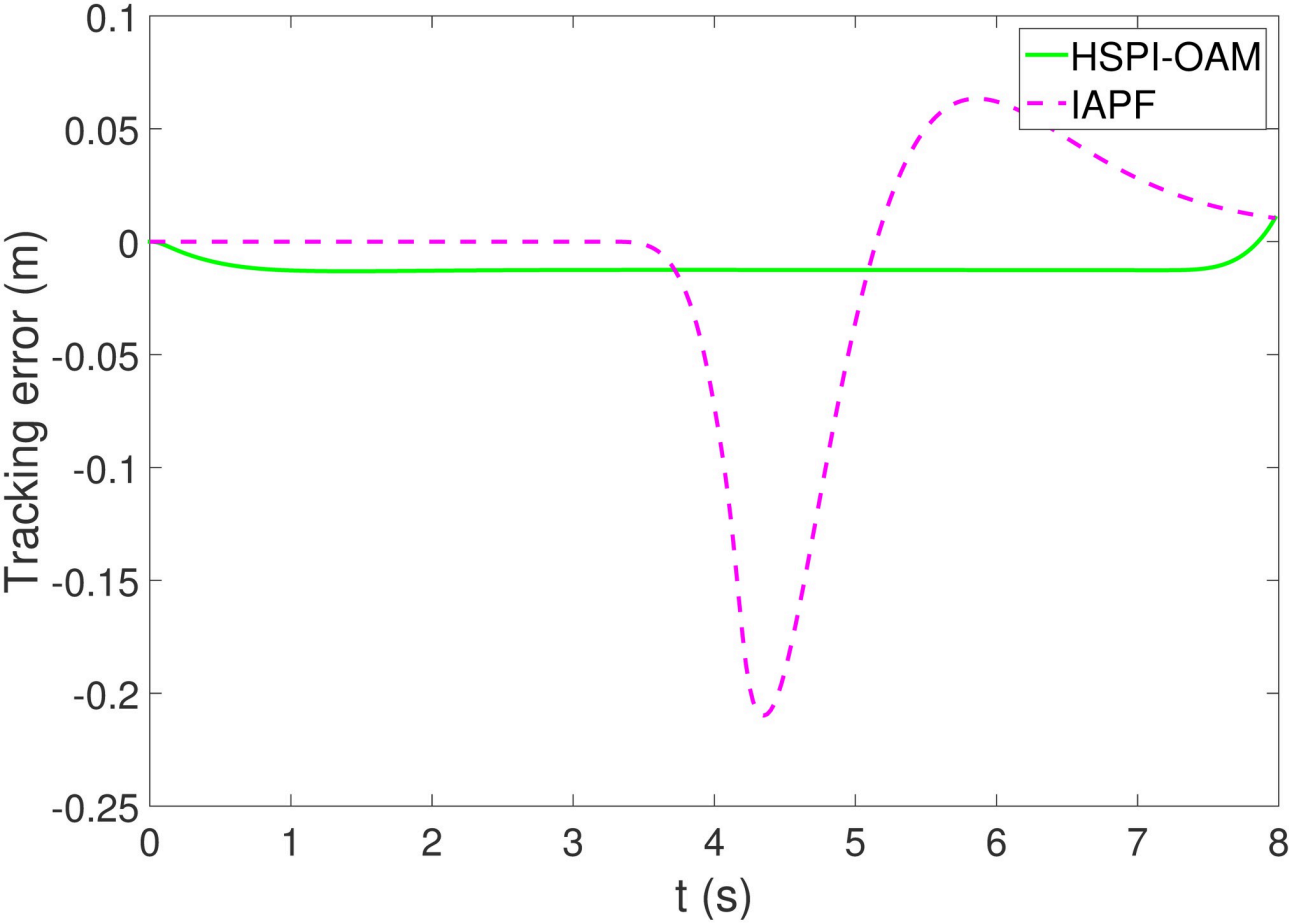
The tracking error of Test 1.

The results of Test 1 are shown in Figs 4–7. In Test 1, the obstacle has only longitudinal acceleration. As shown in Fig 4, The arrow indicates the direction of motion. The area surrounded by the star symbol represents the size of the obstacle. The area surrounded by the red star symbol is the initial position of the obstacle, and the areas surrounded by the star symbol in other colors are the positions of the obstacle when AV is closest to the obstacle in the corresponding method. It is not difficult to see that both methods HSPI-OAM and IAPF can achieve safe obstacle avoidance, while the path in method HSPI-OAM is smoother. In Fig 5, the maximum side slip angle of AV is 0.13^∘^ in HSPI-OAM, while IAPF does have 1.08^∘^. And the yaw rate has the same curve variation trend as shown in Fig 6, which means that the path obtained by HSPI-OAM is very smooth and conducive to vehicle stability, and the path tracking error of AV can also be minimized, as shown in Fig 7.

### Test 2

**Fig 8:**
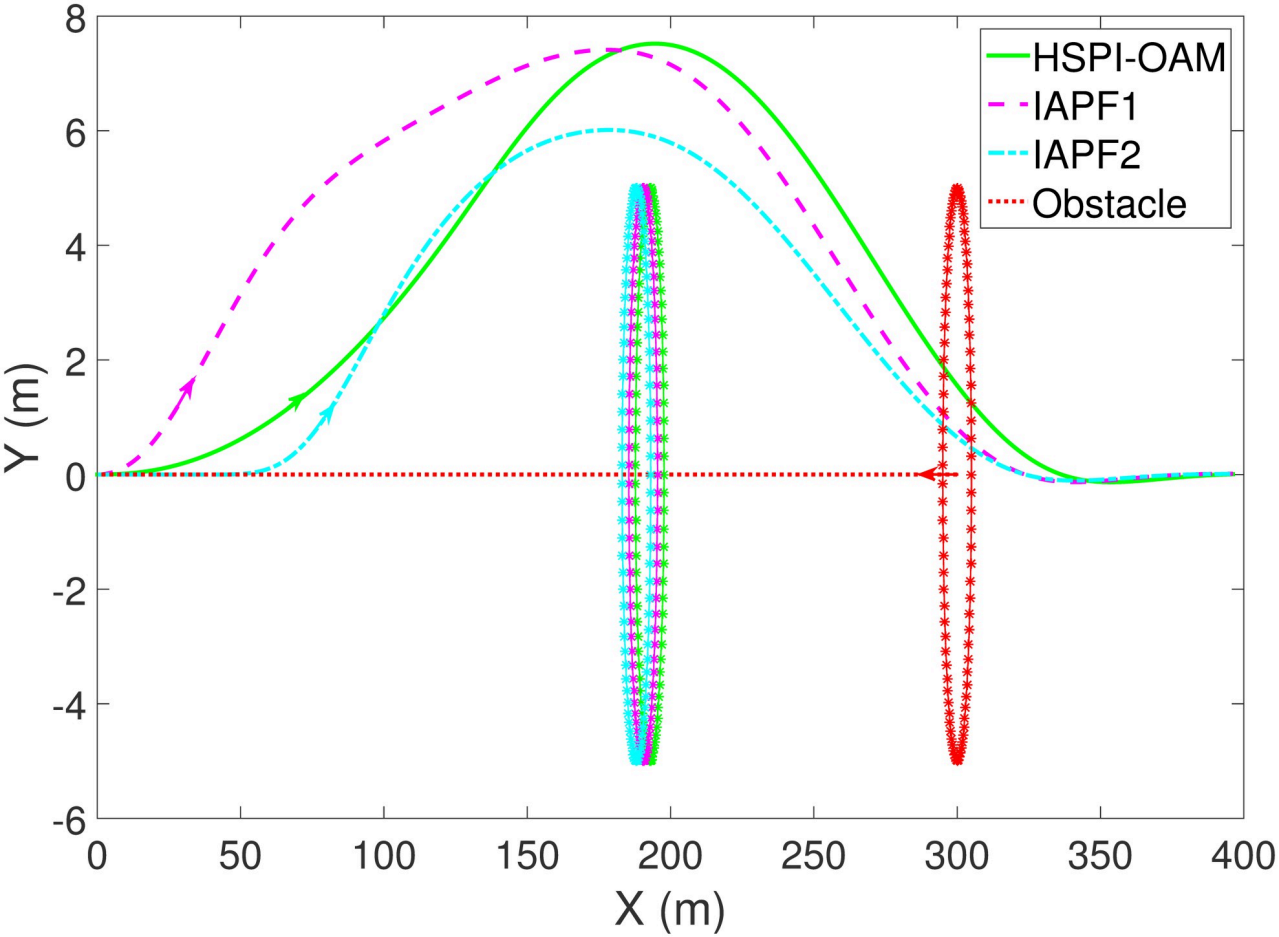
The trajectory of AV and obstacle of Test 2.

**Fig 9:**
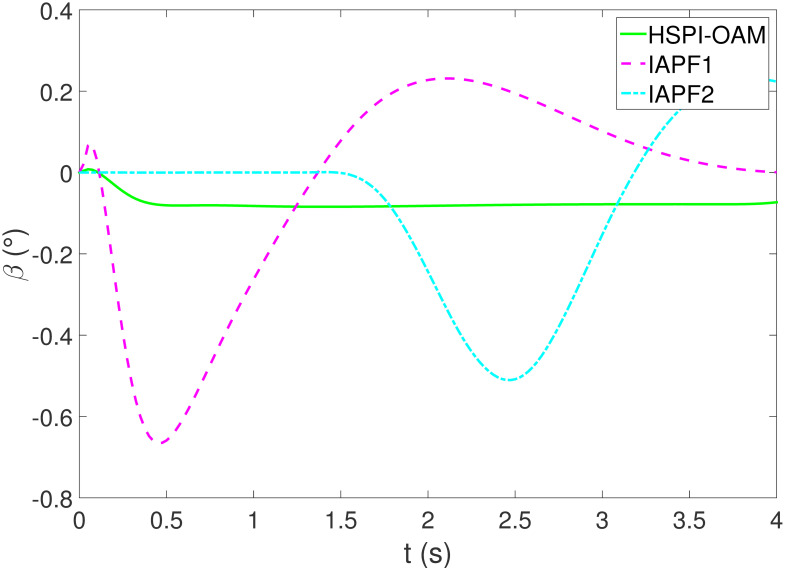
The side slip angle of Test 2.

**Fig 10:**
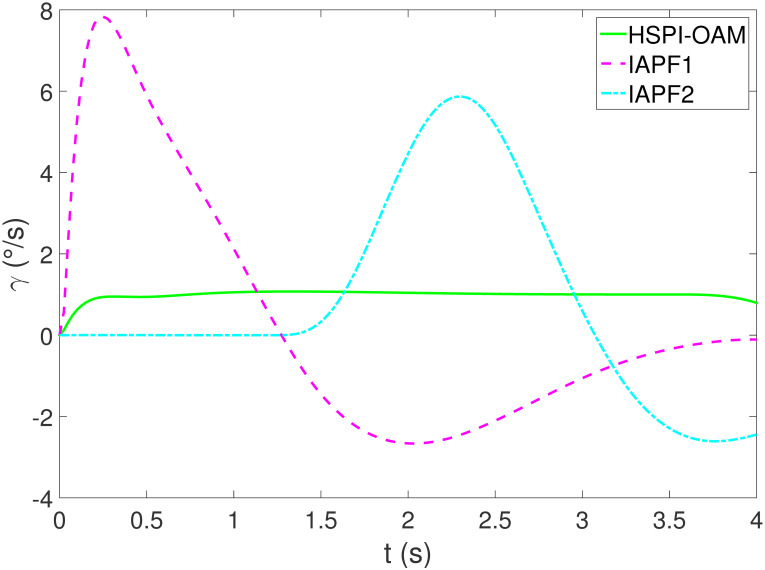
The yaw rate of Test 2.

**Fig 11:**
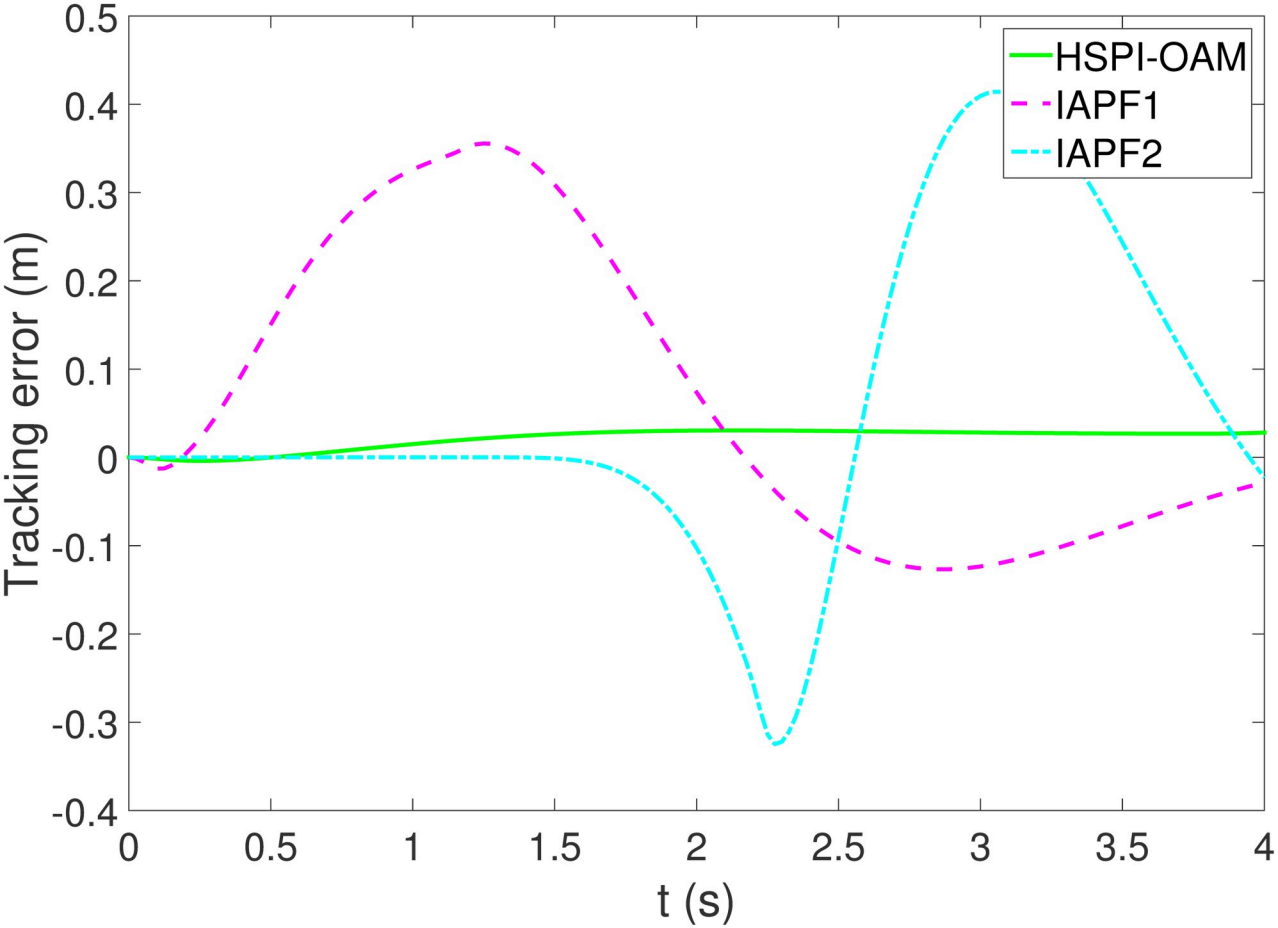
The tracking error of Test 2.

The results of Test 2 are shown in Figs 8–11. Compared with Test 1, the motion parameter in Test 2 is changed. Corresponding to the change of motion parameter, the performance parameter involved in HSPI-OAM does not change as shown in Table 2, while the side slip angle, yaw rate and tracking error of AV remain in a very small range. In IAPF1, the same parameters as in Test 1 are used. Although obstacle avoidance is still completed, the path is indeed greatly changed. In IAPF1, the path implements sudden maneuver for obstacle avoidance, which is not in line with human obstacle-avoidance operation, but also greatly damages the smoothness of the path. In IAPF2, the weight of obstacle avoidance cost is reduced to *k*_1_ = 0.0015, and the path similar to IAPF in Test 1 is obtained. Therefore, the performance of obstacle avoidance in IAPF depends on the choice of parameters, while HSPI-OAM achieves good obstacle avoidance performance in Test 1 and Test 2 by using the same parameters.

### Test 3

**Fig 12:**
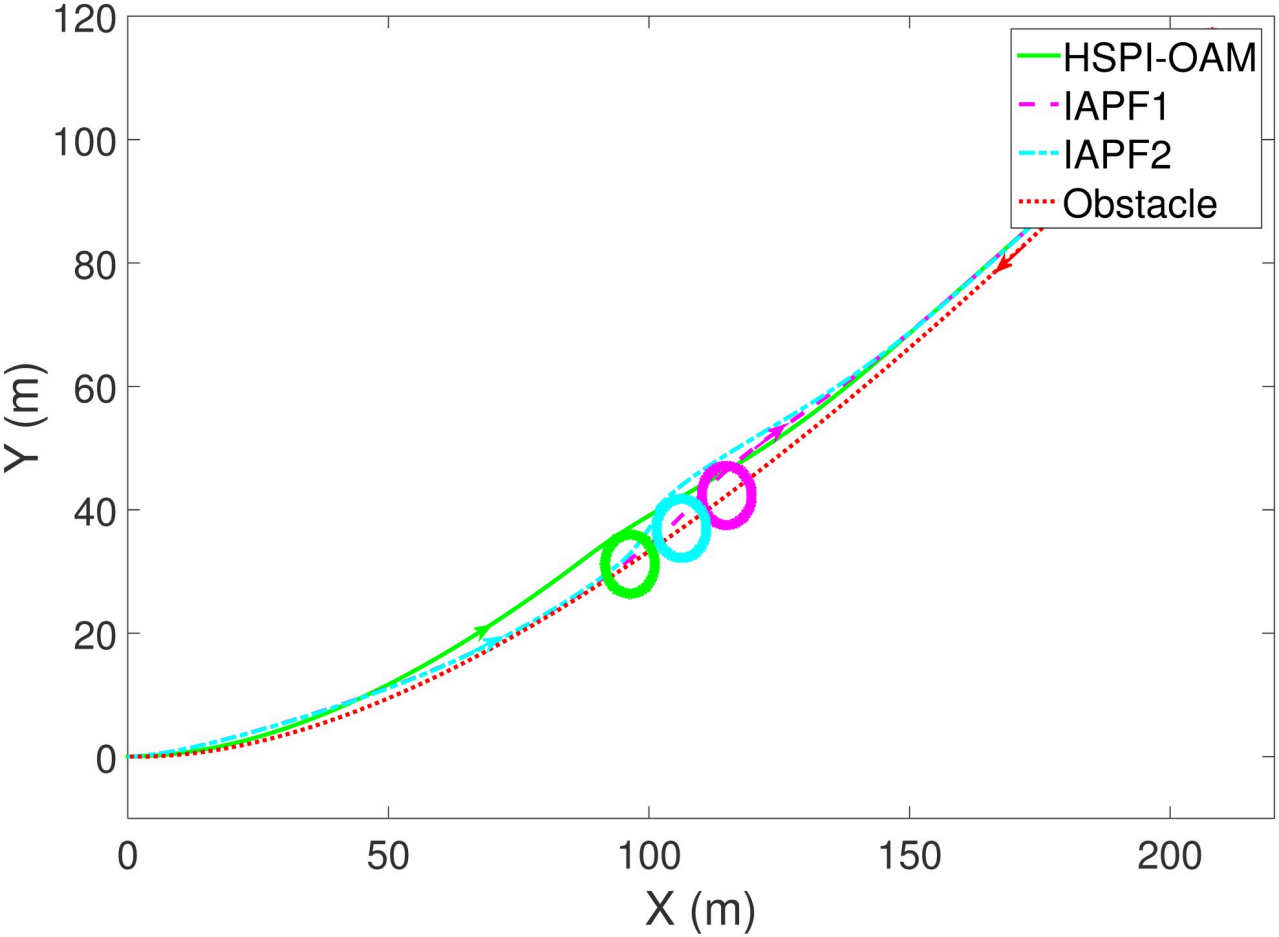
The trajectory of AV and obstacle of Test 3.

**Fig 13:**
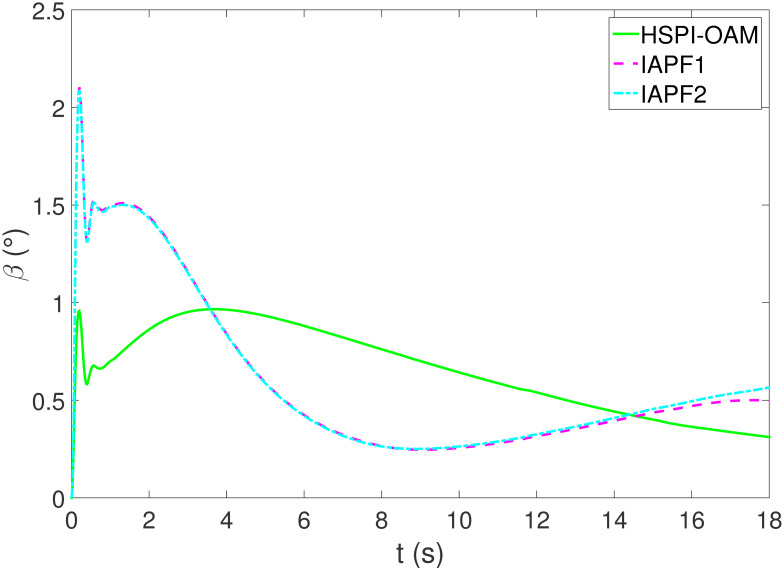
The side slip angle of Test 3.

**Fig 14:**
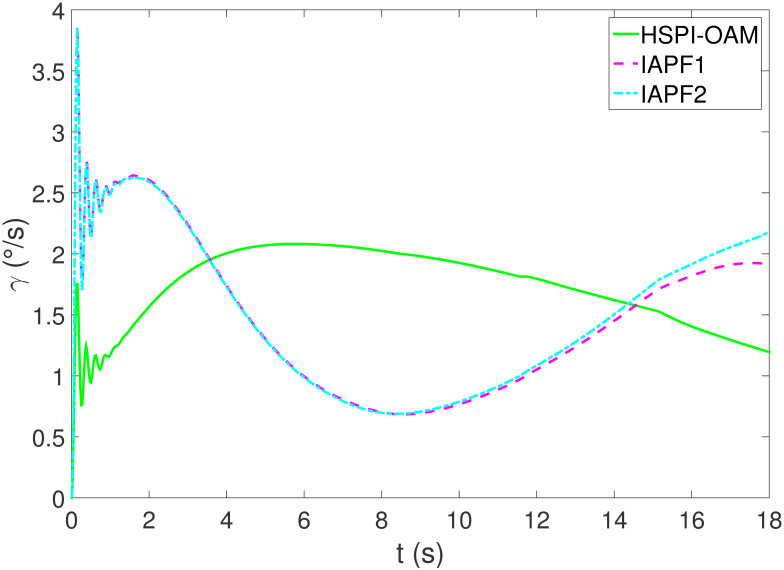
The yaw rate of Test 3.

**Fig 15:**
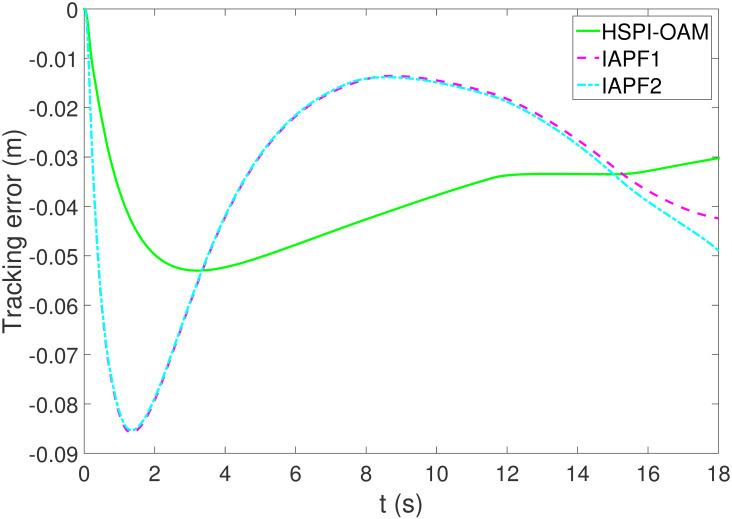
The tracking error of Test 3.

The results of Test 3 are shown in Figs 12–15. Unlike Tests 1 and 2, both obstacle and vehicle in Test 3 have longitudinal and lateral acceleration, so their trajectories are curved. In HSPI-OAM, the performance parameter is still unchanged, and AV still achieves obstacle avoidance while the side slip angle, yaw rate and tracking error remain at fairly small values. *k*_1_ = 0.1 in IAPF1 is the same as that in Test 1. Different from Test 1 and Test 2, the vehicle collides with the obstacle because *k*_1_ = 0.1 value is too small in Test 3. In IAPF2, *k*_1_ is adjusted to *k*_1_ = 1.5 to complete obstacle avoidance again. Not only do IAPF1 and IAPF2 depend on the parameter, but the side slip angle, yaw rate and tracking error in IAPF1 and IAPF2 is greater than those in HSPI-OAM.

### Test 4

**Fig 16:**
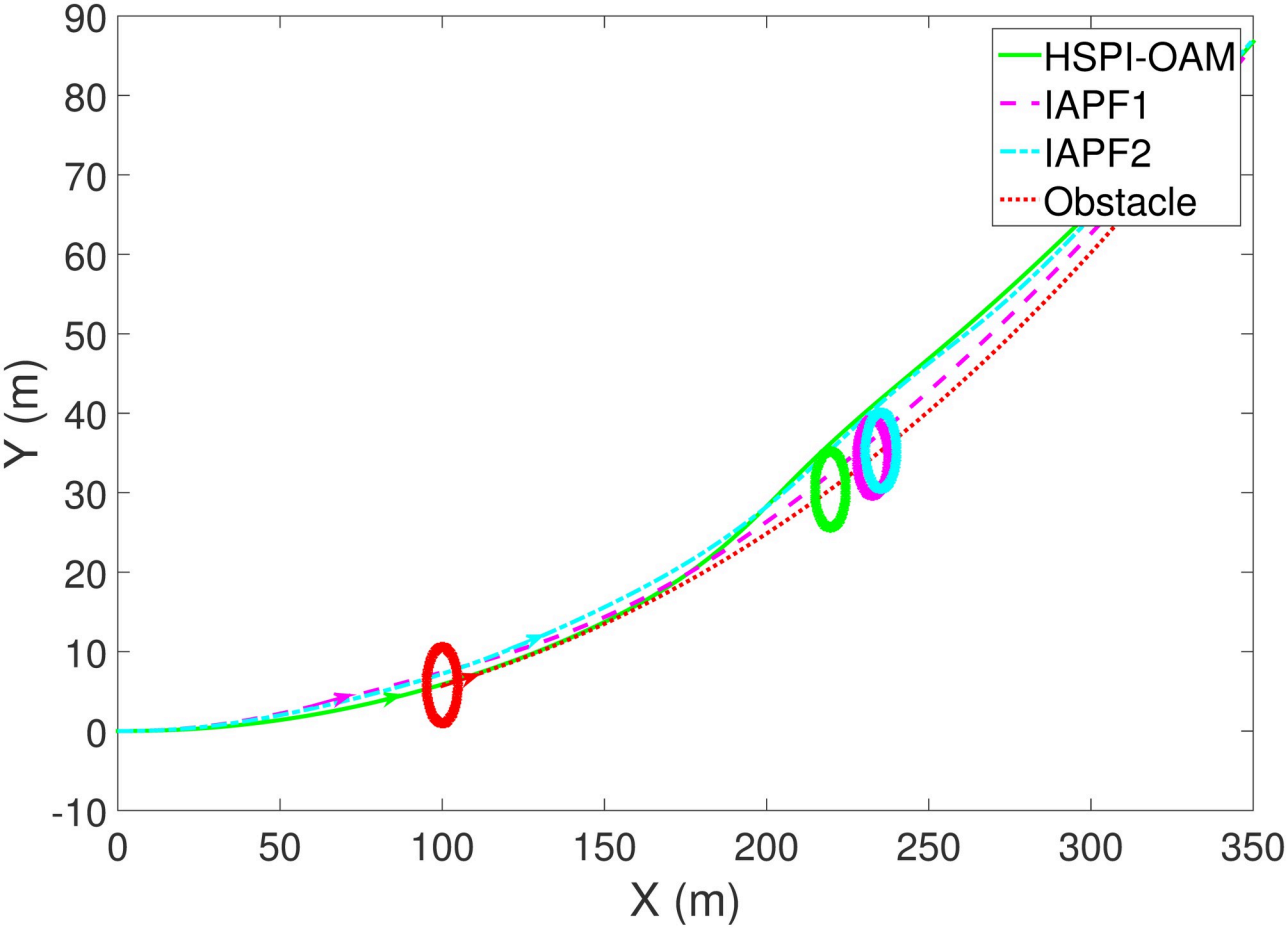
The trajectory of AV and obstacle of Test 4.

**Fig 17:**
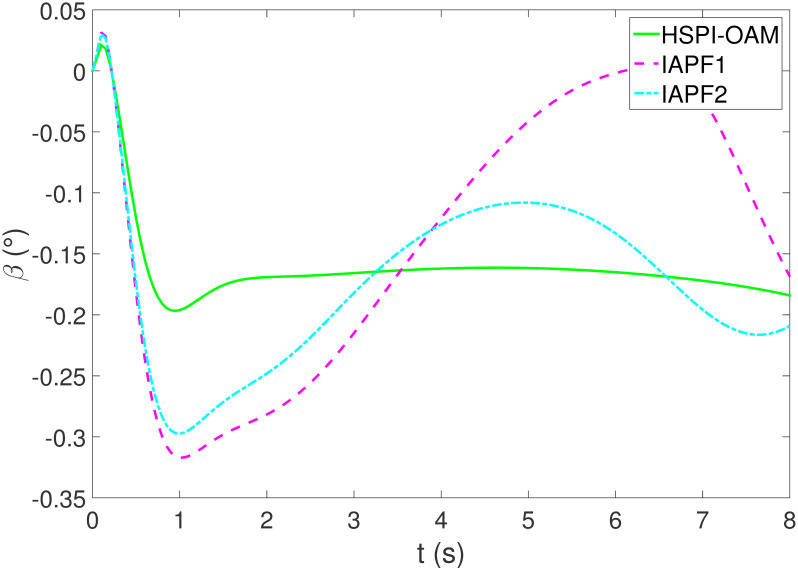
The side slip angle of Test 4.

**Fig 18:**
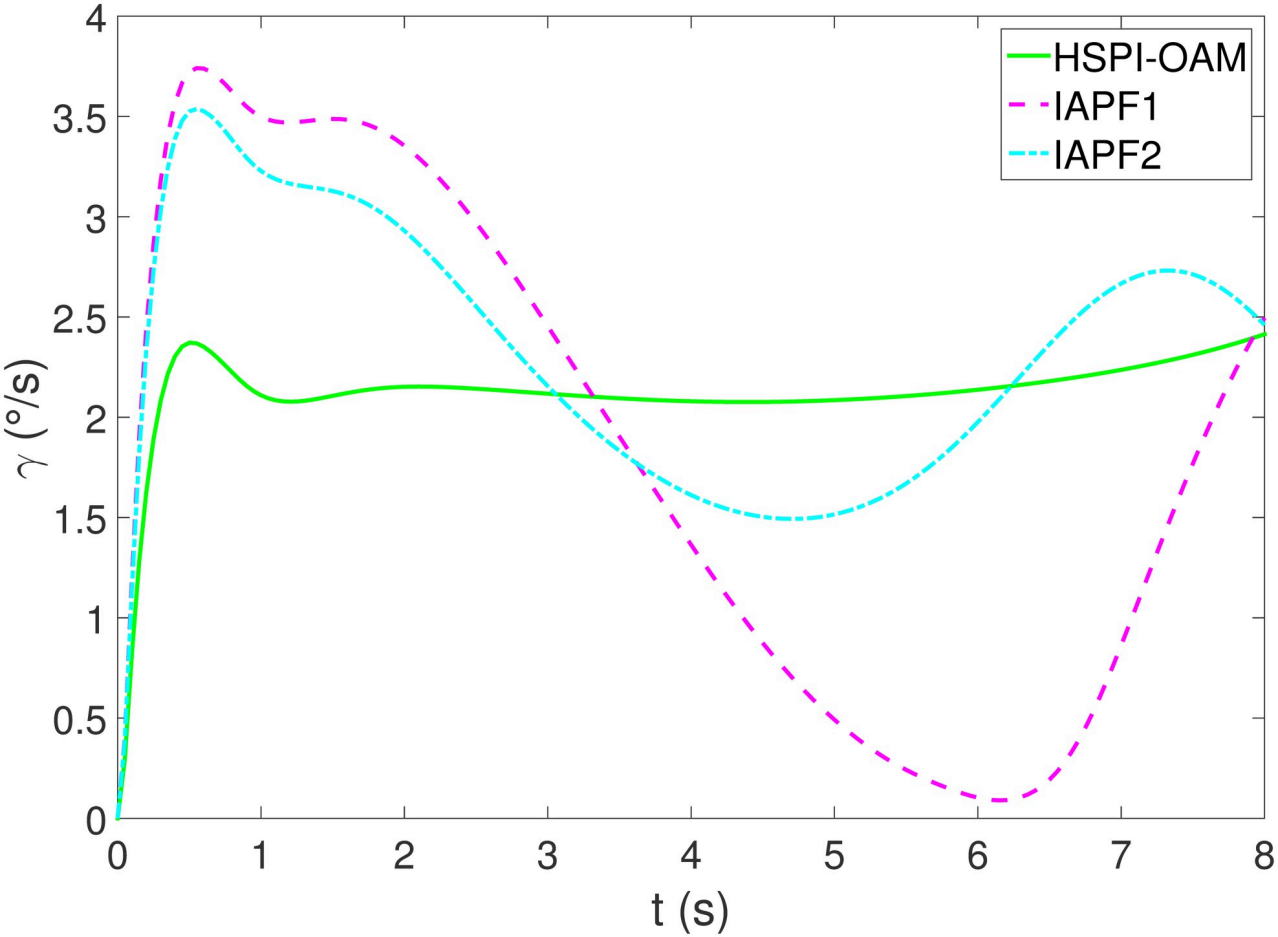
The yaw rate of Test 4.

**Fig 19:**
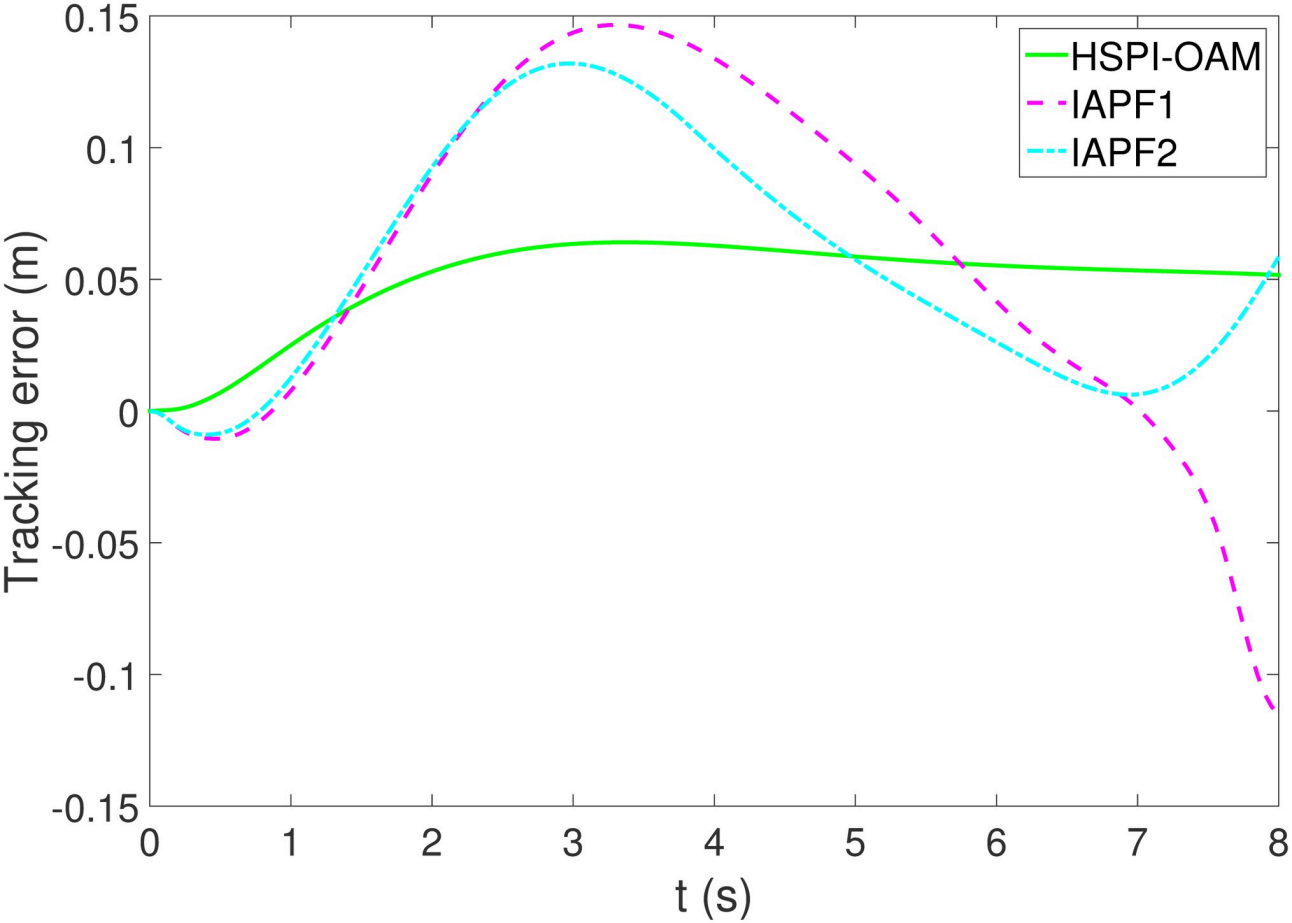
The tracking error of Test 4.

The results of Test 4 are shown in Figs 16–19. As in Test 3, both obstacle and vehicle in Test 4 have longitudinal and lateral acceleration. The difference is that AV and obstacle are moving in the same direction. In HSPI-OAM, the performance parameter is still unchanged, and AV still achieves obstacle avoidance while the side slip angle, yaw rate and tracking error remain at fairly small values. *k*_1_ = 1.5 in IAPF1 is the same as that in IAPF2 of Test 3. While the parameter that can realize obstacle avoidance in IAPF2 of Test 3 has a collision in this working condition. In IAPF2, *k*_1_ = 1.5 is adjusted to *k*_1_ = 50 to avoid collision.

In addition, the comparison method uses the combination of artificial potential field and model prediction method to plan the path. Adjusting the prediction time domain in the model prediction can improve the performance of the comparison method to a certain extent. For example, we use 5, 10 and 15 to as the prediction time. The longer the prediction time domain, the earlier the comparison algorithm can implement the path planning, thus smoothing the path to a certain extent. However, the required cost will also increase. More importantly, the smoothness of its path cannot be compared with that of the proposed method, and the improvement of yaw rate, side slip angle and tracking error of the vehicle controlled by the comparison method is also limited. And the proposed method is planned at the acceleration level and the acceleration is equalized in the virtual collision point model, so the smoothness of the planned path is greatly improved.

## Section 5: Conclusions

The proposed HSPI-OAM can realize highly smooth and parameter independent obstacle avoidance in the complex obstacle avoidance situation where both the obstacle and the vehicle have acceleration. In four different working conditions, the comparison method needs to adjust parameters according to different working conditions to achieve obstacle avoidance, and the smoothness of the path is not as good as HSPI-OAM. HSPI-OAM can achieve obstacle avoidance and obtain a smooth path without adjusting parameter, which is conducive to the stability of the vehicle and the accuracy of path tracking. Since the proposed method accurately designs the required acceleration according to the motion information of the moving obstacle and vehicle, it may be necessary to add additional strategies for pre-processing each obstacle in the face of multiple obstacles, so as to improve the adaptability of the method to the multi-obstacle environment, which is also a direction for the subsequent efforts of the method.

## Supporting information

S1 Data (RAR)
